# Supplementary material for: A Critical View of the Application of the APEX Software (Aqueous Photochemistry of Environmentally-Occurring Xenobiotics) to Predict Photoreaction Kinetics in Surface Freshwaters
Source: Molecules. 2019 Dec 18;25(1):9. doi: 10.3390/molecules25010009 (PMC7017383; doi:10.3390/molecules25010009)
Supplement: Supplementary file 1 [file molecules-25-00009-s001.zip › 3.2.4_gcc-4.4.0.zip!/3.2.4_gcc-4.4.0/3.2.4_gcc-4.4.0/doc/octave/html/liboctave/Collocation-Weights.html]

Collocation Weights - Octave C++ Classes


Previous: Quadrature,
Up: Quadrature


---

### 10.1 Collocation Weights

— : **CollocWt** (void)  
— : **CollocWt** (int n, int inc\_l, int inc\_r)  
— : **CollocWt** (int n, int inc\_l, int inc\_r, double l, double r)  
— : **CollocWt** (int n, double a, double b, int inc\_l, int inc\_r)  
— : **CollocWt** (int n, int inc\_l, int inc\_r, double l, double r)  
— : **CollocWt** (const CollocWt&)

— : CollocWt& **operator =** (const CollocWt&)

— : CollocWt& **resize** (int ncol)

— : CollocWt& **add\_left** (void)  
— : CollocWt& **add\_right** (void)

— : CollocWt& **delete\_left** (void)  
— : CollocWt& **delete\_right** (void)

— : CollocWt& **set\_left** (double val)  
— : CollocWt& **set\_right** (double val)

— : CollocWt& **set\_alpha** (double val)  
— : CollocWt& **set\_beta** (double val)

— : int **ncol** (void) const

— : int **left\_included** (void) const  
— : int **right\_included** (void) const

— : double **left** (void) const  
— : double **right** (void) const  
— : double **width** (void) const

— : double **alpha** (void) const  
— : double **beta** (void) const

— : ColumnVector **roots** (void)  
— : ColumnVector **quad** (void)  
— : ColumnVector **quad\_weights** (void)

— : Matrix **first** (void)  
— : Matrix **second** (void)

— : ostream& **operator <<** (ostream &os, const CollocWt &c)
